# A functional Kv1.2-hERG chimaeric channel expressed in *Pichia pastoris*

**DOI:** 10.1038/srep04201

**Published:** 2014-02-26

**Authors:** Mandeep S. Dhillon, Christopher J. Cockcroft, Tim Munsey, Kathrine J. Smith, Andrew J. Powell, Paul Carter, David C. Wrighton, Hong-lin Rong, Shahnaz P. Yusaf, Asipu Sivaprasadarao

**Affiliations:** 1 School of Biomedical Sciences, Faculty of Biological Sciences; 2 Multidisciplinary Cardiovascular Research Centre, University of Leeds, LS2 9JT, Leeds, U.K; 3 GlaxoSmithKline, Gunnels Wood Road, Stevenage, Hertfordshire, SG1 2NY, UK

## Abstract

Members of the six-transmembrane segment family of ion channels share a common structural design. However, there are sequence differences between the members that confer distinct biophysical properties on individual channels. Currently, we do not have 3D structures for all members of the family to help explain the molecular basis for the differences in their biophysical properties and pharmacology. This is due to low-level expression of many members in native or heterologous systems. One exception is rat Kv1.2 which has been overexpressed in *Pichia pastoris* and crystallised. Here, we tested chimaeras of rat Kv1.2 with the hERG channel for function in *Xenopus* oocytes and for overexpression in *Pichia*. Chimaera containing the S1–S6 transmembrane region of HERG showed functional and pharmacological properties similar to hERG and could be overexpressed and purified from *Pichia*. Our results demonstrate that rat Kv1.2 could serve as a surrogate to express difficult-to-overexpress members of the six-transmembrane segment channel family.

Ion channels represent a major family of membrane proteins, each evolved to perform a specific, often unique, physiological task^1^. Knowledge of high resolution structure for each member of the family would not only help understand the molecular basis for their unique function, but would facilitate structure-guided drug development. Although in recent years, there have been significant breakthroughs in the structural biology of ion channels^2^,^3^,^4^, structures are available only for a handful of channels and the majority of these structures are not for human proteins. The reason for the frustratingly slow progress is that these channels occur in low abundance and many of them are difficult to over-express in heterologous systems in quantities suitable for X-ray crystallographic studies.

One potential approach to circumvent this problem is to generate chimaeric constructs between a channel (we name this ‘host') that has been successfully over-expressed and crystallised and the desired target channel (we name this ‘guest'). An ideal chimaera would include parts of the host channel that enable over-expression of functionally important and ‘druggable' regions of the ‘guest' channel. Such approaches are already yielding structural information on key functional elements of several ion channels^5^,^6^, although they are not of human in origin.

A major subfamily of ion channels is the six-transmembrane (6-TM) segment channels. They are tetramers, each subunit comprising 6 membrane spanning domains with cytoplasmic N- and C-termini. With over 140 members, the family comprises voltage-gated potassium (Kv) channels, cyclic nucleotide gated (CNG) channels, hyperpolarisation activated (HCN) channels and TRP (transient receptor potential) channels^1^. They contribute to a wide range of important physiological roles in the body, including neuronal excitability, muscle contraction, regulation of hormone secretion, vascular tone and several specialised functions^1^. As such, they are being increasingly recognised as novel drug targets for a variety of diseases. The fact that they all share a common membrane topology, and presumably follow a similar folding paradigm, allows design of functional chimaeras between a member whose structure has been solved and a member whose structure is important to solve.

Here we chose the rat Kv1.2 channel as a ‘host' because it is closer to human channels than bacterial channels, has been over-expressed in *Pichia pastoris*^7^ and subsequently crystallised to produce a high resolution structure^8^. We chose the *ether-a-go-go* related (hERG) potassium channel as ‘guest' because this is one of the most sought-after ion channels when it comes to structure. It is of interest to channel biologists because it displays unique biophysical properties and plays a crucial role in cardiac rhythm^2^,^3^,^4^, and to the pharmaceutical industry because it presents a significant hurdle in the way of production of safe drugs (see later^9^).

hERG, encoded by *KCNH2*, contributes to the IK_r_ current which helps to repolarise the cardiac action potential and maintain the cardiac rhythm^2^,^3^,^4^. If its function is compromised, either due to an inherited genetic mutation, or drug blockade, the result is prolongation of the cardiac action potential, a condition described as the Long-QT syndrome (LQTS), which in turn could lead to cardiac arrhythmias and even sudden death^2^,^3^,^4^. hERG mediated sudden death as a side effect of non-antiarrhthmic drugs has been receiving increased attention by the regulatory bodies^2^,^3^,^4^,^9^.

As mentioned above, hERG exhibits unusual biophysical properties. When the cardiac cell membrane is depolarised, it is activated (opening of activation gates) slowly, but undergoes rapid C-type inactivation (closure of inactivation gates). This property allows the channel to remain in a closed state during depolarisation and thereby sustain the plataeau phase of the cardiac action potential^2^,^3^. As the cardiac action potential begins to hyperpolarise (due to activation of IK_s_), the inactivation gate opens rapidly (recovery from inactivation) but the activation gate closes (deactivation) slowly. This allows K^+^ efflux and rapid repolarisation of the action potential. These properties are the reason why hERG displays inward rectification- a property unique to hERG^2^,^3^,^4^.

hERG is blocked promiscuously by a variety of structurally unrelated drugs. This has led to the withdrawal of several blockbuster drugs (e.g. Tefenadine) from the market and to mandatory (FDA requirement) screening of all new drugs for hERG blockade prior to clinical trials. Consequently, it has been recognised that drugs should be screened for hERG blockade prior to clinical trials^3^,^9^.

hERG belongs to K_v_ channel subfamily. Its S1–S4 segments form the voltage sensing domain (VSD) and the S5–S6 together with the P-loop form the pore domain (PD). VSD contains positively charged residues (Arg/Lys) in S4 and three conserved negative charges (Asp/Glu) in S2–S3^2^,^3^,^4^,^10^. In addition, hERG contains three additional negative charges. During depolarisation, S4 moves out of the electric field, leading to the opening of activation gates located at the cytoplasmic end of the pore^2^,^3^,^4^,^10^,^11^,^12^,^13^. The C-type inactivation gate is at the extracellular end of the pore^2^,^3^,^4^,^14^. The N-terminal PAS domain and the C-terminal cyclic nucleotide binding domain (cNBD) accelerate deactivation kinetics^2^,^3^,^4^,^15^,^16^,^17^,^18^.

The aim of the study was to generate a Kv1.2-hERG chimaera that not only retains the unique biophysical and drug binding properties of the hERG channel, but could be overexpressed in *Pichia* in milligram quantities to facilitate structural studies.

## Results

### Generation of a functional Kv1.2-hERG chimaera

**Figure 1:**
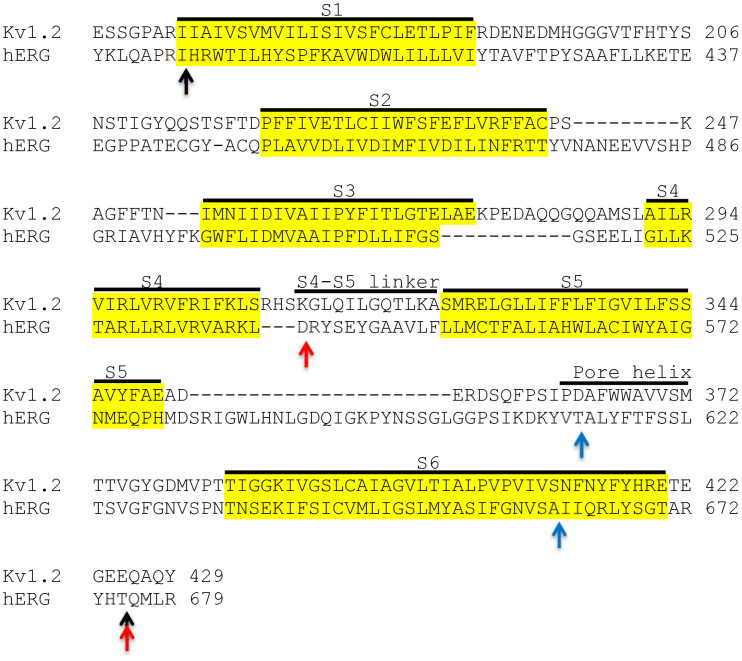
Sequence alignment of Kv1.2 and hERG showing sites of substitutions. Potential transmembrane regions (highlighted in yellow) and other functional elements (S4–S5 linker and pore-helix) are labelled. Arrows indicate sites where different chimaeras were joined: Black, S1–S6; red, S4/S5–S6; blue, P-S6.

**Figure 2:**
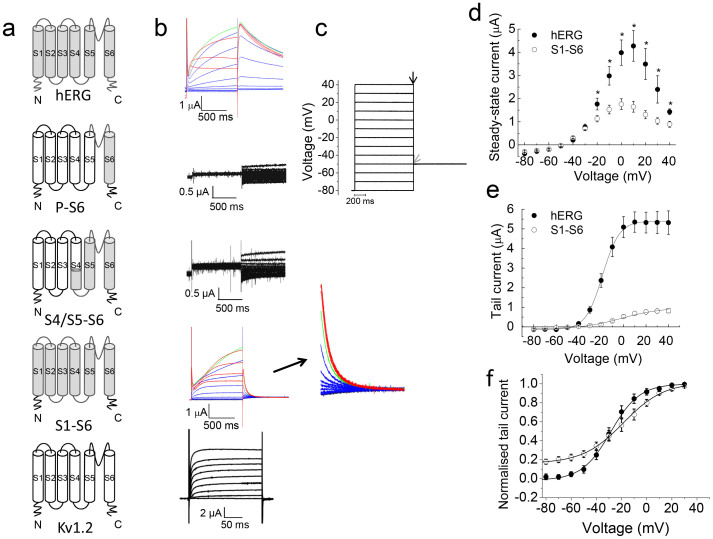
Functional analysis of hERG-Kv1.2 chimaeras. (a) Schematic of constructs showing hERG segments (gray) substituted into Kv1.2 (black and white) (b) Representative current families recorded from oocytes injected with cRNA corresponding to the constructs in (a). Currents for the chimaeric channels were recorded using the hERG voltage step protocol (−80 mV to +40 mV in 10 mV intervals, followed by a step to −50 mV; interpulse interval was 20 s). Currents through K_V_1.2 were recorded at various step potentials (−80 mV to +80 mV) delivered in 10 mV increments from a holding potential of −80 mV; interpulse interval was 10 s. Current traces for hERG and the S1–S6 chimaera are shown in colour (hERG: −80 to 0 mV, blue; +10 mV, green; +20 to +40 mV, red; and S1–S6 chimaera: −80 to −10 mV, blue; 0 mV, green; +10 to +40 mV, red). Also shown is the scaled version of tail currents for the S1–S6 chimaera. (c) Schematic of the protocol used to record currents through hERG and the chimaeras. (d) Plots of voltage against steady-state currents (mean ± SEM) recorded at the time point indicated by a black arrow in C. * indicates currents through hERG are significantly greater than the S1–S6 chimaera (p < 0.05). (e) Plots of voltage against peak tail currents (mean ± SEM) recorded at the beginning of the −50 mV pulse (corresponding to gray arrow in c). The lines joining the mean currents represent Boltzmann distribution. The V_1/2_ value of the S1–S6 chimaera (−10.78 ± 3.55 mV, n = 13) is not significantly different (p > 0.05) from that of hERG (−18.03 ± 0.90 mV, n = 20). However, slope (k) values are significantly different (p < 0.05) between hERG (6.99 ± 0.22, n = 20) and the S1–S6 chimaera (12.94 ± 1.66, n = 13). (f) Normalised plot of data from (e).

Despite significant progress in our understanding of the structure-function relationships of hERG^2^,^3^,^4^ and recent breakthroughs in the structural biology of Kv channels^6^,^8^, our knowledge is still inadequate to allow *in silico* design of chimaeric channels that are certain to give rise to functional channels. Likewise, we do not know which regions of Kv1.2 are essential to drive high level expression in *Pichia*, while supporting the function of the substituted domains. For this reason, we have taken an empirical approach and generated three chimaeras by substituting different functional domains of hERG into Kv1.2 in the hope that one or more of these chimaeras would give rise to functional channels and can be over-expressed in *Pichia*. In designing the chimaeras, we were guided by the Kv1.2-hERG sequence alignment (Fig. 1), generated on the basis of the studies of Kv1.2^8^ and KvAP^19^,^20^, and mutagenic studies on the functional regions of hERG^10^,^11^,^21^. The chimaeras were named P-S6, S4/S5–S6, and S1–S6; the names correspond to the regions of hERG substituted into Kv1.2 (Fig. 2a). P-S6 harbours the selectivity filter, drug binding sites and the S6 channel gate. S4/S5–S6 has additional domains that couple the voltage sensor to pore gating. The third chimaera, S1–S6 has the entire transmembrane portion of hERG. This would be an ideal construct because if functional, its structure would explain what makes hERG unique in terms of both its biophysical and drug binding properties.

To test the function, we expressed the chimaeric constructs in *Xenopus* oocytes and measured currents using the two-electrode voltage clamp technique (Fig. 2b). Currents were elicited by a series of depolarising pulses, delivered at 10 mV intervals, to +40 mV from a holding potential of −80 mV, followed by repolarisation to −50 mV (hERG). Both P-S6 and S4/S5–S6 constructs failed to show currents either during the depolarising steps or subsequent repolarisation (Fig. 2b). By contrast, S1–S6 displayed currents that were comparable to hERG (Fig. 2b,d). Like hERG, the S1–S6 chimaera activated slowly at potentials positive of −50 mV; current amplitudes increased with voltage (voltage dependent activation), but at more positive voltages, the amplitudes declined due to inactivation (Fig. 2b,d). The current amplitudes of the chimaera, however, were consistently smaller (~3-fold) than those of hERG for an equivalent amount of injected cRNA.

### Comparison of voltage dependence of activation of the S1–S6 chimaera with hERG

The voltage dependent properties of the chimaera are markedly similar to those of hERG (Fig. 2d–e). When currents (I) at the end of each test pulse (steady-state currents) were plotted against the voltage (V) applied, a bell-shaped relationship was apparent for both hERG and the chimaera (Fig. 2d); the decline in current amplitude at positive potentials is due to inactivation, a feature unique to hERG. Furthermore, the chimaera passed significant current (tail current) during the −50 mV repolarising voltage step (Fig. 2b,e,f), indicating slow closure (deactivation, coupled with rapid recovery from inactivation- see later) of the chimaeric channel. This again is a hallmark feature of hERG. In Fig. 2e, peak tail currents (at −50 mV) were plotted against the preceding voltage pulse using the Boltzmann function to obtain biophysical parameters that determine the voltage dependence of channel activation. Activation of the chimaeric channel occurred at potentials positive of −50 mV, similar to the hERG channel. The V_1/2_ (voltage for half-maximal activation) value (−10.78 ± 3.55 mV) is not significantly different (P > 0.05) from that of hERG (−18.03 ± 0.9 mV). The voltage dependence of channel activation (slope factor, k = 12.94 ± 1.66 mV) of the chimaera, however, is significantly greater (P < 0.05) than that of hERG (k = 6.99 ± 0.22 mV).

Taken together, these results suggest that substitution of S1–S6 of hERG confers on Kv1.2 the key biophysical properties of the hERG channel, including slow activation, strong voltage dependent inactivation and slow deactivation. The minor differences in biophysical parameters are expected because the chimaera lacks the cytosolic domains of hERG, which are known to modulate the biophysical properties conferred by the transmembrane domain^2^,^3^,^4^,^15^,^16^,^17^,^22^,^23^.

### Comparison of activation and deactivation properties of the S1–S6 chimaera with hERG

**Figure 3:**
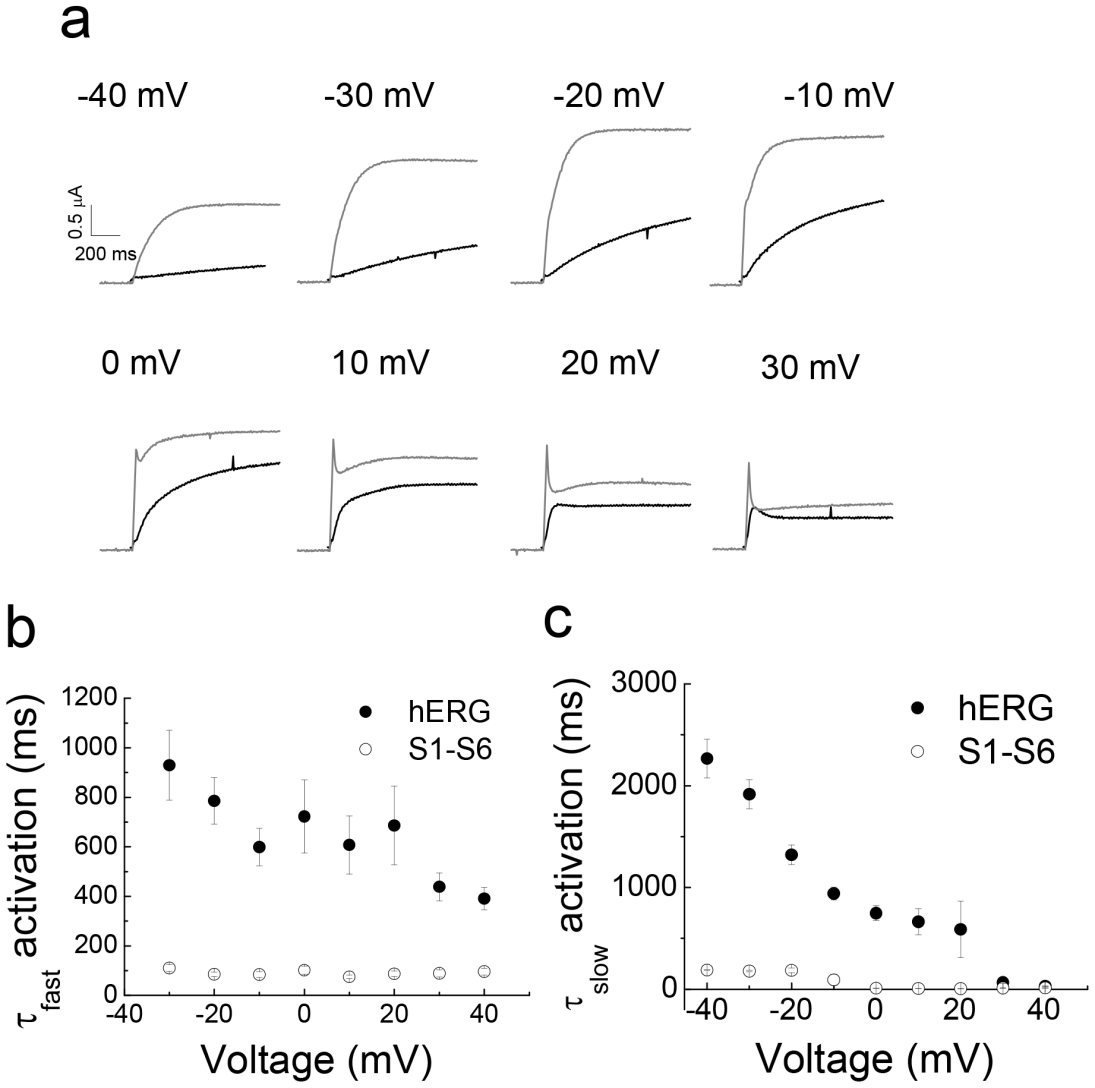
Comparison of the activation kinetics of hERG and the S1–S6 chimaera. (a) Comparison of representative channel activation current traces between hERG (black traces) and the S1–S6 (grey traces) chimaeras at the indicated voltage steps. (b–c) Comparison of the voltage dependence of fast (b) and slow (c) activation time constants calculated from the activation current traces, as described in methods (chimaera, n = 8; hERG, n = 5). The fast as well as slow activation kinetics of the S1–S6 chimaera are significantly faster than those for hERG (p < 0.05) at all potentials examined.

The time-course of activation for the S1–S6 chimaera at various membrane potentials, is compared with the superimposed hERG activation curves in Fig. 3a (chimaera: grey traces; hERG: black traces). The results indicate that the rate of activation of hERG and the chimaera is voltage dependent; the activation curves could be described with fast and slow activation time constants. However, the activation kinetics of the chimaera, both fast and slow, are significantly faster than that of the hERG channel at all potentials between −40 mV and 20 mV (Fig. 3b,c). These results indicate that the cytosolic domains of hERG play a role in the slow activation of hERG. Interestingly, such faster kinetics were described for erg1-sm, a smooth muscle isoform of hERG, which lacks the C-terminal 101 amino acids, but is otherwise very similar to the cardiac isoform^24^.

Besides faster activation kinetics, the chimaera displayed large transient currents at potentials positive to 0 mV, the amplitude of which increased with the increase in depolarisation. Although hERG current traces show occasional transients, they are considerably smaller, being detectable only at strong depolarising potentials (≥30 mV). In this respect, again, the chimaera is similar to erg1-sm which showed large transient currents^24^. Such large transients are also a characteristic feature of ELK channels^25^, which are closely related to hERG.

**Figure 4:**
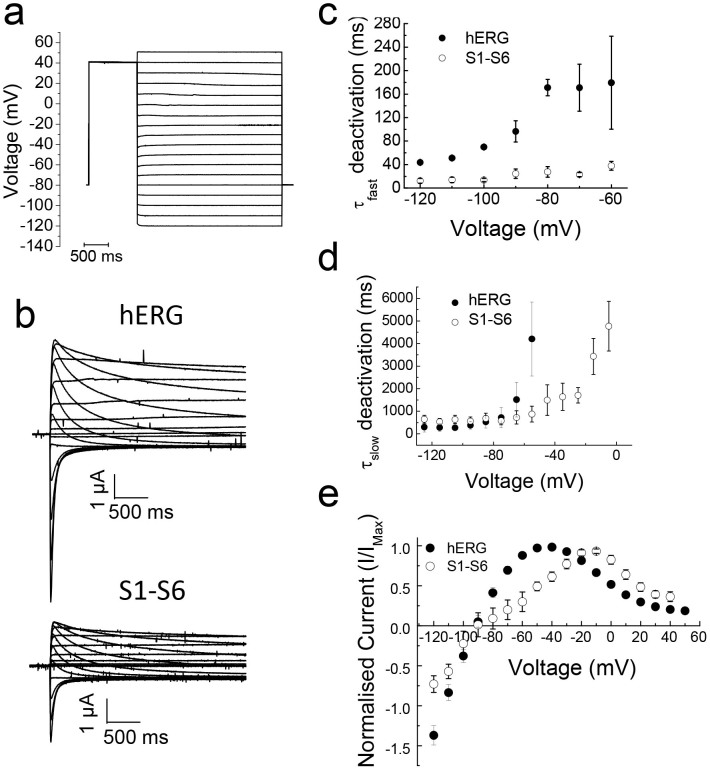
Comparison of the deactivation kinetics of hERG and the S1–S6 chimaera. (a) Voltage protocol used to measure deactivation kinetics. (b) Representative deactivation current traces of hERG and the S1–S6 chimaera at various voltage potentials following the +40 mV pulse. (c–d) Comparison of the voltage dependence of fast (b) and slow (c) deactivation time constants calculated from deactivation current traces, as described in methods (chimaera, n = 8; hERG, n = 5). The fast deactivation kinetics of the S1–S6 chimaera are significantly faster than those for hERG (p < 0.05). (e) Normalised current-voltage relationships of peak fully activated currents (n = 7). E_rev_ values were calculated by fitting the linear section of the curve and calculating the x-intercept (not shown). E_rev_ values were ≈−90 mV.

Deactivation kinetics were determined from current traces recorded at various repolarisation potentials (−120 to +50 mV) following a strong depolarising pulse (+40 mV; Fig. 4a). Fig. 4b shows representative deactivation current traces during repolarisation for hERG and the S1–S6 chimaera. The traces could be described with slow and fast components, from which time constants for deactivation were calculated for each voltage. Fig. 4c–d shows plots of repolarising voltage against fast and slow deactivation time constants. The results show that deactivation kinetics of the S1–S6 chimaera for the fast component are significantly faster than those of hERG. At −90 mV, for example, the mean τ_fast_ (time constant for fast deactivation) for the chimaera was 24.5 ± 8.1 ms, and that for hERG was 96.4 ± 18.0 ms. Time constants (τ_slow_) for the slow (minor) component of deactivation showed no significant difference between hERG and the chimaera in the voltage region (−120 to −60 mV) where they could be reliably determined. In this respect, the chimaera again shows similarity to erg1-sm, which deactivates about four fold faster than hERG^24^.

### Comparison of inward rectification properties of the S1–S6 chimaera with hERG

**Figure 5:**
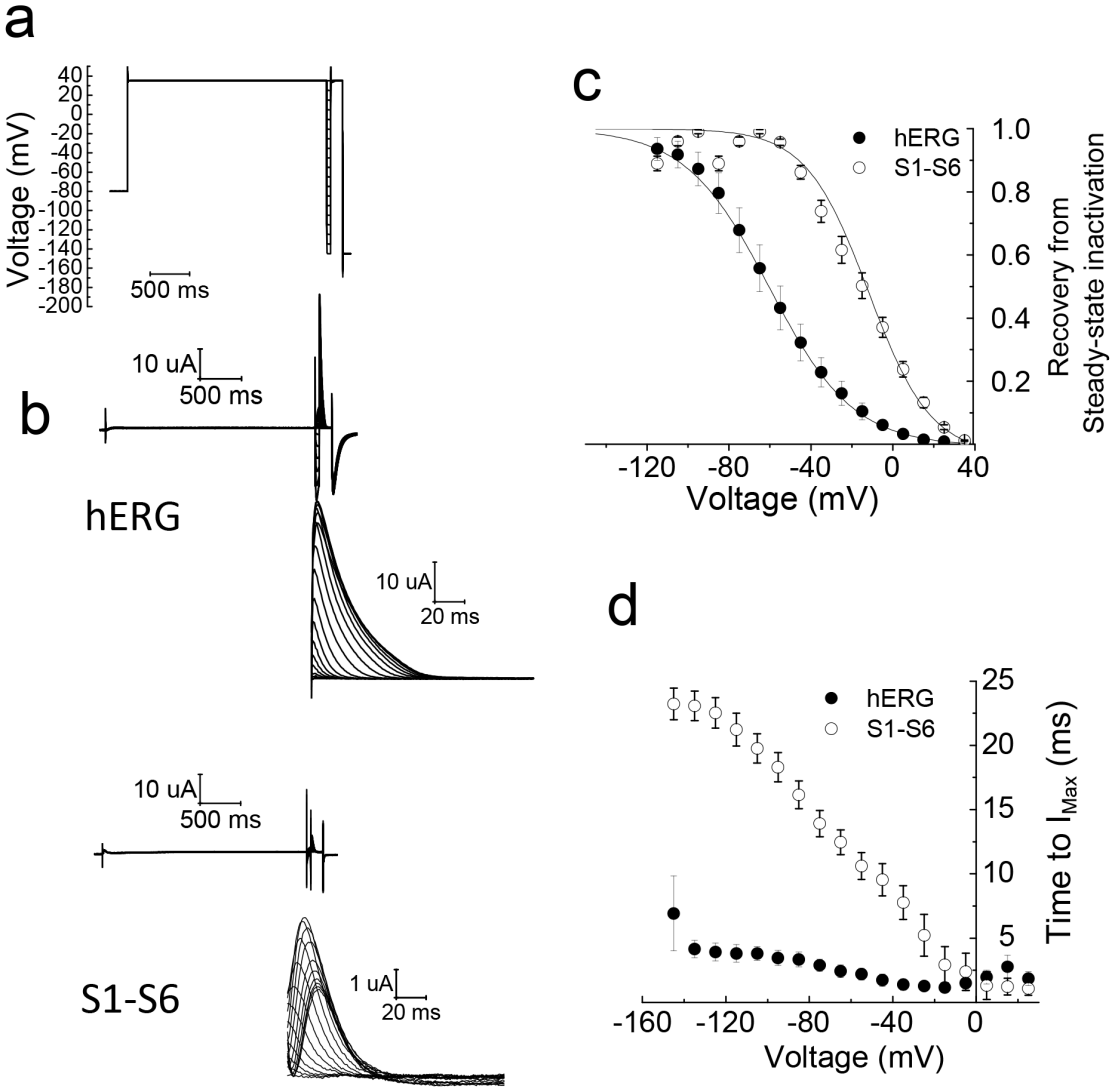
Comparison of recovery from inactivation of hERG and the S1–S6 chimaera. (a) The three stage voltage protocol used to measure recovery from inactivation. Voltage was first stepped from −80 mV to + 35 mV to fully inactivate the channels, then stepped from −145 to +35 mV, in 10 mV increments, followed by a third +35 mV step, and then a final −145 mV step to relieve inactivation. (b) Representative current traces for hERG and the chimaera using the protocol shown in (a). The scaled-up traces show currents during the third +35 mV step; they represent the currents recovered from inactivation during the preceding voltage steps. (c) Voltage-dependence of the recovery from steady-state inactivation. The normalised peak recovered currents were plotted against voltage and fitted with a Boltzmann function. There was a significant (p < 0.05,) rightward shift in the V_1/2_ value of ≈40 mV for the S1–S6 chimaera (−17.09 ± 2.37, n = 12) compared to hERG (−60.47 ± 6.20 mV). (d) Voltage dependence of the time to reach peak current (I_Max_); the S1–S6 chimaera takes significantly (p < 0.05) longer time compared with hERG to reach peak current over the voltage range examined.

hERG is unique among Kv channels in that it shows inward rectification. The mechanism of inward rectification in hERG, however, is mechanistically different from that of inwardly rectifying potassium (Kir) channel family^2^,^3^,^4^. In hERG, inward rectification is brought about by fast inactivation at depolarising potentials followed by the rapid recovery from inactivation, coupled with slow deactivation, at hyperpolarised potentials. Fig. 4e shows a plot of peak current amplitudes during the repolarising pulses against voltage for hERG and the S1–S6 chimaera (protocol Fig. 4a). Both hERG and the chimaera display bell-shaped I-V relationships, with reversal potentials (~−90 mV) expected for a potassium selective channel. However, there is a rightward shift for peak current amplitudes by ~20 mV (from −40 mV for hERG to −20 mV for the chimaera). This shift appears to result from a positive shift in activation (Fig. 2e) and even greater shift in inactivation (Fig. 5c, see later).

### Comparison of inactivation properties of the S1–S6 chimaera with hERG

A standard three pulse protocol was used to determine the voltage dependence of inactivation/channel availability (Fig. 5a). A 1 s pulse to +35 mV was first applied in order to attain steady state inactivation. This was followed by a series of test pulses to potentials ranging from −145 to +35 mV for 20 ms which is long enough to allow recovery of channels from inactivation, but too short to allow deactivation. The third pulse to +35 mV was to measure the relative number of channels that have recovered from inactivation during the preceding second pulse. Fig. 5b shows representative currents from the protocol, alongside currents recorded during the third pulse. Although both the chimaera and hERG show currents recovered from inactivation, there are striking differences between the two. As demonstrated previously^26^, hERG displays instantaneous currents at the start of the third pulse; these currents represent channels that have fully recovered from inactivation, but failed to undergo deactivation during the preceding test pulse. Like hERG, the chimaera also showed currents from channels that have recovered from inactivation; the kinetic profile of these currents, however, is different from that of hERG. The kinetics of recovered chimaeric channel currents are markedly dependent on the potential in the preceding test pulse; the more negative the voltage of the preceding pulse was, the slower were the kinetics. The time required to reach peak currents (I_Max_) as a function of preceding test voltage is depicted in Fig. 5d. One possible explanation for this result is that where the test pulse potentials are very negative, some of the channels might not only have recovered from inactivation, but have undergone deactivation. During the third pulse, such channels would be activated slowly resulting in a slow onset of currents. Thus whereas with hERG, currents during the third pulse represent mostly currents that have recovered from inactivation, with the chimaera, the currents are due to recovery from inactivation as well as from activation of deactivated channels. The more negative the test potential is, the greater would be the population of deactivated channels. Such explanation is consistent with the faster deactivation kinetics of the chimaera relative to hERG (Fig. 4). An alternative explanation is that the channel goes through several closed states before opening, the population of which is voltage dependent. Such properties results in Cole-Moore shift (where the rate of activation depends on the preconditioning pulse), which is exhibited by the eag channels (KCNH1) that are closely related to hERG^27^.

Peak currents elicited at the beginning of the third pulse were plotted as a function of the variable voltage of the second pulse using the Boltzmann function. The results (Fig. 5c) show a large positive shift in voltage required for recovery from inactivation of the chimaera (−17.09 ± 2.37 mV for chimaera, compared to hERG −60.47 ± 6.20 mV). A similar positive shift in inactivation has been reported for erg1-sm^24^.

### Comparison of dofetilide binding properties of the S1–S6 chimaera with hERG

**Figure 6:**
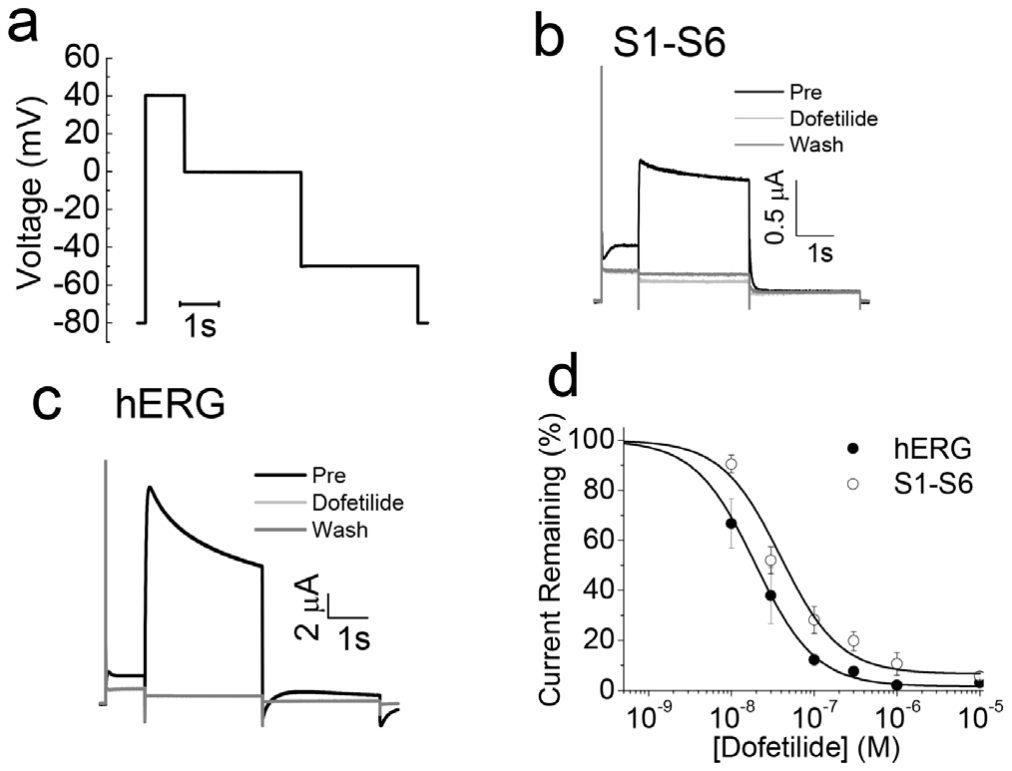
The S1–S6 chimaera is inhibited by dofetilide, the classical hERG pore blocker. (a) A three step voltage protocol used to determine inhibition by dofetilide. (b–c) Representative current traces for hERG (b) and the S1–S6 chimaera (c) before (black trace) and during (light gray trace) application of 10 μM dofetilide and after washout of the drug (dark gray). (d) Dofetilide concentration-response relationship for hERG and the S1–S6 chimaera. IC_50_ value for inhibition by dofetilide for the S1–S6 chimaera (39.54 ± 8.68 nM) was not significantly different from that for hERG (19.32 ± 9.6 nM) (p > 0.05; n = 4).

Dofetilide, a class III anti-arrhythmic drug is a potent blocker of hERG and is widely used to distinguish hERG currents from other currents^9^,^28^. For this a three step voltage protocol as outlined in Fig. 6a was used. Application of 10 μM dofetilide for 10 min caused complete inhibition of both the chimaera and the hERG channel (Fig. 6b–d). To examine if there is any difference in the affinity of the drug between the chimaera and hERG, effect of increasing concentrations of the drug on current inhibition was determined. The results (Fig. 6d) show that there is no significant difference (*p* > 0.05) in the IC_50_ (drug concentration at which 50% of the channels are blocked) values between the chimaera (39.54 ± 8.68 nM) and the hERG channel (19.32 ± 9.6 nM).

### Overexpression of hERG in *Pichia*

**Figure 7:**
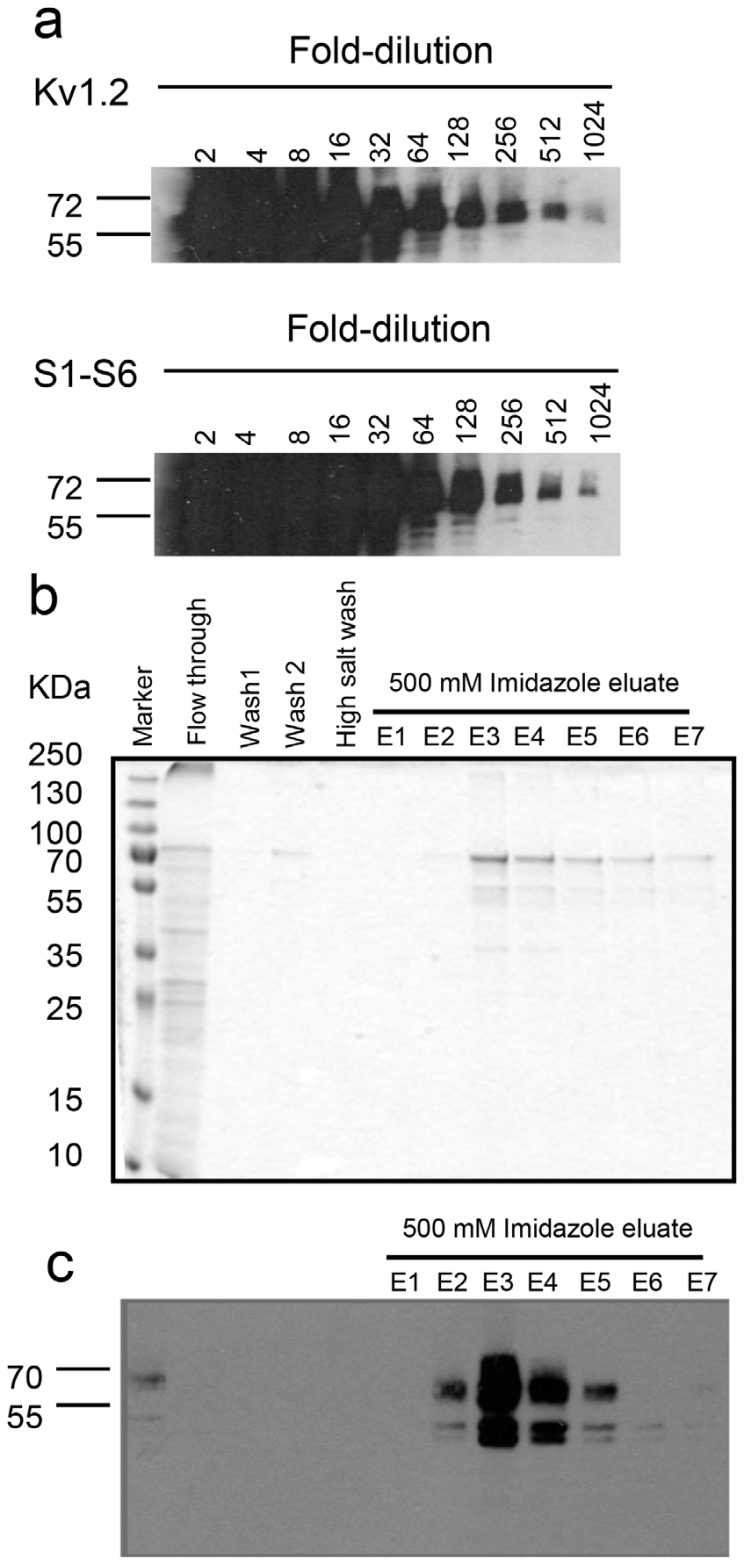
Expression and purification of the S1–S6 channel protein in *Pichia pastoris*. (a) Western blots of Kv1.2 and the S1–S6 chimaera. Methanol induced cells pelleted from 1 ml culture (at a density of O.D. 8.0) were extracted into 200 μl of SDS sample buffer using glass beads and serial dilutions of the extract were subjected to western blotting using anti-His antibodies (b) Purification of the S1–S6 chimaera by Talon metal affinity chromatography. 45 mg of solubilised membrane proteins (input) were subjected to purification as described in methods. E1–E7 represent fractions eluted with 500 mM imidazole. 5 μl of each fraction were subjected to SDS-PAGE and Coomassie blue staining. (c) Western blotting of diluted fractions from the affinity purification. The input (lane 1) and flowthrough were loaded at a dilution of 1:50, whilst all the other samples were loaded at 1:10 dilution.

Having demonstrated that the chimaeric channel exhibits the key functional and pharmacological properties of the channel, we next set out to express the chimaeric channel in *Pichia pastoris* (it may be noted that we were unable to express hERG in *Pichia*). For this we cloned the cDNA encoding the His-tagged S1–S6 chimaera into the pPIC3.5 *Pichia* expression vector and used the resultant construct to transform the KM71 strain of *Pichia pastoris*. To compare the levels of expression, we have also transformed the cells with pPIC3.5-Kv1.2 (Kv1.2 is also His-tagged). Cultures of the transformed cells were treated with methanol to induce the expression of the channels and cells pelleted from 1 ml culture (at a density of O.D. 8.0) were extracted into 200 μl of SDS sample buffer and serial dilutions of the extracts were subjected to western blotting using anti-His antibodies (Fig. 7a). Two bands, corresponding to ~70 KDa and ~60 KDa, were seen in the diluted lanes, which may correspond to fully- and core -glycosylated proteins. Presence of bands at 1:512 and 1:1024 diluted samples with comparable intensities demonstrates that the expression level of the chimaera may be comparable to that of Kv1.2.

We next attempted to purify the S1–S6 chimaera by affinity chromatography using the cobalt resin. Fig. 7b shows Coomassie gel (10% SDS-PAGE) staining of proteins obtained during fractionation of the detergent solubilised membranes (45 mg of membrane protein). A major band around 70 KDa was seen with some low molecular weight bands in lanes eluted with 500 mM imidazole. The minor bands could include breakdown products of the chimaera, as can be seen in the western blot of corresponding fractions, where the major band is in the region of 70 kDa (Fig. 7c).

## Discussion

Rat Kv1.2 is the only member of the eukaryotic 6-TM mammalian channels whose structure could be determined by X-ray diffraction^8^. Progress in the determination of other members of this family is hampered largely by the inability to overexpress these channels in amounts sufficient to generate crystals. The reasons why some proteins can be overexpressed, but others are poorly expressed in heterologous systems are not known, although one contributing factor that has been shown to determine protein expression is the N-terminus^29^. Furthermore, smaller proteins can be expressed relatively more easily than large complex proteins. For these reasons, we postulated that the N-terminus of rat Kv1.2 could be used to support overexpression of the transmembrane domains of other 6-TM channels. A key requirement, however, is that the chimaeras are functional and display the properties of the substituted domain. It is well known that the majority of the functional properties in 6-TM channels are associated with their transmembrane domains. Furthermore, it is generally assumed that folding of these domains is conserved despite the differences in their amino acid sequences. Thus we hoped to generate chimaeras that could not only be overexpressed but are functional. To test our hypothesis, we chose hERG as a candidate ‘guest' channel because its sequence is significantly different from that of Kv1.2 (~90% different in the transmembrane portion)^15^,^16^,^17^ and exhibits unusual functional properties and pharmacology. In particular, it is promiscuously blocked by structurally diverse drugs- which is a common reason for removal of drugs from the market and for failure of drugs to enter clinical trials^3^,^9^.

Here, we report successful generation of a Kv1.2 chimaera that contained the S1–S6 transmembrane portion of hERG. It not only exhibited the functional and pharmacological properties of hERG, but could be overexpressed in *Pichia pastoris*. Co-incidentally, the study also provided important insights into the structure-activity relationships of hERG.

The other two chimaeras, S4/S5–S6 and P-S6, were not functional when expressed in *Xenopus* oocytes. Swartz and colleagues have reported extensive studies on chimaeras in which they substituted segments of KvAP^30^, or TRP channels (TRPM8 and TRPV1)^31^ into Kv2.1. Of the many chimaeras they studied, three of their pore chimaeras between Kv2.1 and KvAP (C_3_(S5–S6)AP, C_7_(S5–S6)AP and C_11_(S5–S6)AP) are comparable to our S4/S5–S6 chimaera (see Supplementary figure 1 in Alabi et al^30^). Their data showed that substitution of residues beyond the S6 helix results in loss of function. Our S4/S5–S6 chimaera also had a substitution beyond the S6 helix. Although this may seem consistent with their results, the same substitution in our S1–S6 chimaera had no effect on function. Thus the reasons for lack of function for the S4/S5–S6 chimaera remain unclear. The study by Alabi et al did not include chimaeras comparable to our P-S6.

The S1–S6 chimaera harbours the major functional domains of hERG: the pore, the voltage sensor, activation and inactivation gates and drug binding pocket. All properties attributed to these domains were largely borne by the chimaera, including slow activation (Figs. 2–3), slow deactivation (Fig. 4), fast inactivation (Figs. 2, 5), strong inward rectification (Fig. 4) and inhibition by the class III anti-arrhythmic drug, dofetilide (Fig. 6). However, there are interesting differences in the parameters that govern the biophysical properties of the hERG channel: (i) the activation and deactivation kinetics of the chimaera are relatively faster than hERG, (ii) the chimaera showed fast (large) transient currents, the amplitude of which increased with depolarisation, and (iii) the amplitudes of currents elicited during repolarisation (tail currents) are much smaller than hERG. Intriguingly, much of the biophysical behaviour of the channel is rather more similar to erg1-sm, a smooth muscle isoform of hERG^24^ than to hERG.

Many of the differences between erg1-sm and hERG have been attributed to a single position in the S4 segment. hERG has a valine at 535, whereas erg1-sm has an alanine at the equivalent position, A537^24^. Mutation of alanine to valine (A537V) in erg1-sm converted many of its biophysical properties to become similar to those for hERG, including the loss of transients, larger outward tail currents upon repolarisation and slow activation. Conversely, when valine in hERG was mutated to alanine, the resulting mutant, V535A, displayed transient currents, smaller tail currents and faster deactivation kinetics. In fact, properties of the V535A hERG mutant channel seem very similar to that of our S1–S6 chimaera, although our S1–S6 chimaera has valine at position 535. It has been reported that rat erg3, a neuronal isoform of erg, exhibits properties similar to erg1-sm^24^; however, it has a valine at position 537. Collectively, these results suggest that the S4 residue alone (valine vs alanine) does not determine the differences between hERG and erg1-sm and that cytosolic domains play a role. The fact that the S4 positions in hERG are likely accessible to the cytosolic domains^10^ supports such possibility.

The N- and C-termini of hERG, as well as of other isoforms of erg, bear the PAS and cNBD domains respectively^2^,^3^,^4^,^15^,^16^. These domains are thought to influence both deactivation and inactivation kinetics of hERG. A role for C-terminus in activation, roles for both N- and C-termini in deactivation and inactivation have been reported^2^,^3^,^4^,^32^.The Kv1.2 cytosolic domains appended to the S1–S6 chimaera lacks these domains, yet the chimaera exhibits slow kinetics of activation and deactivation, fast inactivation and inward rectification, although they are somewhat attenuated when compared with hERG. These results suggest that the transmembrane portion of hERG is largely responsible for all the unique biophysical properties of the channel and that the cytosolic domains have a modulatory role.

In contrast to hERG, to recover from inactivation the chimaera does not require strong hyperpolarised potentials (V_1/2_ for recovery from steady-state inactivation is ~40 mV positive when compared with hERG) (Fig. 5). This difference may explain the transient activation currents as well as the profile of currents during the final step in the 3-step protocol used to study the steady-state inactivation (Fig. 5). Slower rate of inactivation was suggested as the cause of transients in erg3^33^. During the final step of the steady-state inactivation measurements, the chimaera showed currents that were reminiscent of the Cole-Moore shift reported in eag channels, where residues 7–12 (RRGLVA) of the N-terminus are thought to contribute to this phenomenon^34^. This sequence is absent in the corresponding region of Kv1.2. Thus the origin of the Cole-Moore shift in the chimaera remains unclear, but is likely associated with the transmembrane portion of hERG. One possible, albeit simplistic, biophysical explanation for the Cole-Moore like shift seen with the chimaera is as follows (Supplementary Figure 1): During the first depolarising pulse (see Fig. 5a), the channels open (O) from the closed state (C) and undergo inactivation (I) (C → O → I); during the following second hyperpolarising pulse, the channels recover from inactivation (I → O), but a significant proportion of the open channels also undergo deactivation (I → O → C); during the third depolarising test pulse, open channels are expected to elicit instantaneous outward currents, but the closed channels open slowly (C → O) due to slow activation kinetics. The net result of these events would be appearance of currents that resemble the Cole-Moore shift. In the case of hERG, deactivation is relatively slow; thus the occurrence of I → O → C transition during the second pulse is expected to be limited; for this reason, hERG shows mostly instantaneous currents.

There is very little sequence similarity between the cytosolic domains of hERG and Kv1.2. The sequence homology within the transmembrane regions of the two proteins, likewise, is also low, compared to other members of the Kv channels^2^. Thus the fact that we were able to generate functional chimaeras of Kv1.2 that reproduced almost all properties of hERG is highly significant because it suggests that the transmembrane portion of hERG and cytosolic domains of Kv1.2 could fold into independent functional domains, and folding and function are not dependent on each other. This observation suggests that the approach could be used as a general strategy to generate functional chimaeric channels with other 6-TM channels, including those that are distantly related to Kv1.2.

The S1–S6 chimaera showed expression levels that were comparable to those of rat Kv.2 when examined by western blotting of the extracts of induced *Pichia* cells diluted serially (Fig. 7). Western blotting of the purified fraction showed two bands that presumably correspond to the core and glycosylated versions of the protein. The chimaera showed double bands of similar size when expressed in HEK293 cells and showed clear expression at the plasma membrane, indicating that the chimaera likely attains native conformation (Supplementary Figure 2). However, the functional integrity of the purified chimaera remains to be determined.

In summary, our study demonstrated that the transmembrane region harbouring the key functional elements of hERG can be substituted into Kv1.2 to generate chimaeras that were able to recapitulate almost all of the properties of hERG. Thus Kv1.2 could provide a perfect framework for functional substitution of distantly related Kv and other 6-TM channels. Since Kv1.2 is also one of the eukaryotic 6-TM proteins that could exceptionally be overexpressed and crystallised, the present study presents a promising strategy for overproduction of other 6-TM channels that are difficult to overexpress and crystallise.

## Experimental procedures

### Materials

pPIC3.5-rat Kv1.2 containing the His_9_ tag at the N-terminus^7^ and hERG cDNA in pSP64 were kindly provided Dr. D. Parcej and Prof. S.A. Goldstein respectively. KM71 *Pichia pastoris* cells were from Invitrogen. Enzymes for molecular biology were purchased from New England Biolabs, Promega or Stratagene. All general chemicals were obtained from Sigma Chemicals Co. Mouse anti-His tag, goat anti-mouse HRP were purchased from Novagen and BioRad laboratories respectively.

## Methods

### Preparation of DNA constructs

Overlap extension or substitution PCR was used to generate the desired chimaeric constructs depicted in Fig. 2a. The constructs were made from His_9_-Kv1.2 and hERG cDNA templates, such that all constructs contained the N-terminal His_9_ tag. Three chimaeric constructs were generated using the Kv1.2 and hERG sequence alignment (based on the X-ray structure of Kv1.2 and structure-function studies of hERG) as a guide (Fig. 1). The chimaeras have the following amino acid sequences (hERG in bold; the numbers correspond to the primary sequences of rat Kv1.2 (GI:1235594) and hERG (GI:60391379).

P-S6 1–363/**614–667**/412–499

S4/S5–S6 1–328/**539–675**/425–499

S1–S6 1–164/**395–675**/426–499

The chimaeras were subcloned into the pKS-Globin (Kv1.2, P-S6 and S4/5-S6 constructs) or pBF (hERG and S1–S6) oocyte expression vectors for electrophysiological studies in *Xenopus* oocytes or pPIC3.5 for overexpression in *Pichia pastoris*. All constructs were fully sequenced to confirm the identity.

### Preparation of cRNA and current recordings

cRNA was prepared using the T7/SP6 mMessage machine synthesis kit (Ambion). cRNA was injected into the stage V or VI oocytes, isolated from *Xenopus* toads (euthanised by cervical dislocation after anaesthetization with MS-222) as described previously^11^. Injected oocytes were incubated in ND96 solution (NaCl 96 mM, KCl 2 mM, MgCl_2_ 1 mM, HEPES 5 mM, CaCl_2_ 1.8 mM, pH 7.5, 50 μg/ml G418) at 18°C, for 1–3 days, prior to current recordings. Currents were recorded from oocytes by two-electrode voltage clamp. The recording chamber was perfused with Ringer's extracellular solution (NaCl 115 mM, KCl 2.5 mM, CaCl_2_ 1.8 mM, HEPES 10 mM, pH 7.2) at a rate of ~1 ml/min. Voltage clamp was established with two thin-walled borosilicate glass (GC100F-15, Harvard Apparatus Ltd) microelectrodes filled with 3 M KCl that had a resistance of 0.5 to 5 MΩ. Membrane potential was controlled using a GeneClamp 500 amplifier (Molecular Devices), digitised using a NI USB-6211 (National Instruments) and recorded using WinWCP (V 4.0.5) software. Recordings were filtered at 2 kHz and sampled at 4 kHz. A holding potential of −80 mV was used unless otherwise stated. Protocols used varied depending on the objective of the experiment (see figures).

Currents were elicited by pulsing to various test potentials (−80 mV to +40 mV) for 1 s from a holding potential of −80 mV (Fig. 2c) before stepping to −50 mV (1 s). Currents at the end of the test pulse (steady-state) and at the beginning of the −50 mV step (tail current) were plotted against the test potentials. Activation time constants were determined from the rising phase of currents for each test potential. To determine voltage dependence of fully activated channels, a +40 mV pulse (1 s) was delivered from a holding potential of −80 mV before stepping to various test potentials (−120 to +50 mV) (Fig. 4a). Currents at the beginning of each test potential were plotted against voltage. Deactivation time constants were determined from the decay of currents at each test potential. To determine recovery from steady-state inactivation, a three pulse voltage protocol was used (Fig. 5a). The first +35 mV pulse (1 s) was delivered from a holding voltage of −80 mV to fully inactivate the channels; the second pulse to various test potentials in 10 mV increments (−145 mV to +35 mV) was brief (20 ms) to allow voltage dependent recovery from inactivation; and the third pulse was to +35 mV for 20 s, which allowed measurement of currents recovered during the second pulse. Peak currents during the third pulse were plotted against voltage to determine the voltage dependence of recovery from steady-state inactivation. All data are collected from n ≥ 3.

### Electrophysiological data analysis

Raw data were analysed using Clampfit 9.2 (Molecular Devices) and then compiled in Excel 2007 (Microsoft) and OriginPro 7.5 (Originlab). Mean and SEM values were calculated for current recordings and the non-paired two-tailed Students T-test was used to determine statistical significance where appropriate. Tail currents and recovery from steady state inactivation currents were fitted with unconstrained Boltzmann function (equation 1). 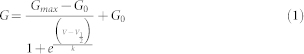
Where G_0_ is the initial conductance, V_1/2_ is the half-activation voltage and k is a measure of the voltage dependence.

Activation time courses were fitted in WinWCP software with a two exponential equation (equation 2). 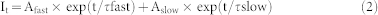
where τ_fast_ and τ_slow_ are the time constants for the fast and slow components of activation, and A_fast_ and A_slow_ are the current amplitudes of each component.

Deactivation time courses were fitted in WinWCP software with two decaying exponentials (equation 3). 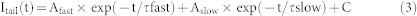
where τ_fast_ and τ_slow_ are the time constants for the fast and slow components of deactivation, A_fast_ and A_slow_ are the current amplitudes of each component, and C is a constant.

Percentage inhibition of current by dofetilide (I_%_) was calculated by normalisation of current in the presence of the drug (I) to current in the presence of vehicle (*I*[B]_0_) (equation 4) 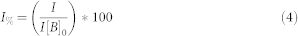
Data from dose dependent drug inhibition of currents were fitted individually to the normalised data for each oocyte with maxima asymptoted to 100% with equation 5. 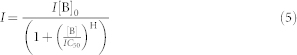
where H is the Hill coefficient, [B] is inhibitor concentration and *I*[B]0 is the current in the absence of inhibitor. IC_50_ values were collated and averaged (±SEM) to determine final IC_50_ values for each construct.

### Expression and purification of the S1–S6 chimaera

Transformation of *P. pastoris* KM71, cell culture and methanol induction of protein expression were carried out as described previously^7^. Colonies resistant to 1 mg/ml G418 were used for protein expression. Cells were grown in 2 l flaks to sufficient density in MGYH (1.34% yeast nitrogen base, 1% glycerol, 0.00004% biotin, 0.004% histidine) medium at 27°C in a shaking incubator (200 r.p.m.) Protein expression was induced by replacing the MGYH medium with MMH (1.34% yeast nitrogen base, 0.5% methanol, 0.00004% biotin, 0.004% histidine) medium and growing cells for 3 days at 27°C in a shaking incubator at 200 r.p.m. 0.5% methanol was supplemented every 24 hr. Cells were harvested by centrifugation and the pellets were suspended in lysis buffer (50 mM phosphate buffer, pH 8.0, 5% (v/v) glycerol, 1 mM EDTA, 34 μg/ml PMSF, 1× EDTA-free protease inhibitor cocktail (Roche)). The suspension was homogenised with 0.5 mm acid washed glass beads in a BeadBeater (BioSpec Products: Bartlesville, USA), following the instructions of the manufacturer. Unbroken cells and glass beads were collected by centrifugation at 4000 g. The supernatant was then centrifuged at 100000 × *g* to pellet the membrane fraction. The membrane pellet was washed by resuspending in lysis buffer and re-centrifugation at 100000 × *g*.

Membranes (50–100 mg) were solubilised in 1% NP-40, 50 mM Tris, 150 mM KCl, 10 mM 2-mercaptoethanol, pH 7.5 at room temperature for 3 hr, using a rotating mixer (detergent to protein ratio was kept at 5:1). The suspension was centrifuged at 100,000 × g for 1 hr to remove insoluble material. The supernatant was loaded onto Talon® metal affinity resin (Clontech) equilibrated with equilibration buffer (0.5% NP-40, 50 mM Tris, 150 mM KCl, 10 mM 2-mercaptoethanol, 20 mM imidazole, pH 7.5). After washing with the equilibration buffer and a high salt wash buffer (1 M NaCl in equilibration buffer), the bound protein was eluted with 500 mM imidazole made up in the equilibration buffer. Aliquots of each fraction were subjected to SDS-PAGE and stained with Coomassie Brilliant Blue.

#### Western blotting

Expression of the S1–S6 chimaera in *Pichia* was detected by western blotting using anti-His-tag antibody (1:20000), goat anti-mouse-HRP conjugated IgG (1:40000), and SuperSignal West Femto Maximum Sensitivity ECL Substrate (Pierce).

## Author Contributions

M.D., C.J.C., P.C., D.C.W., H.L. and T.M. performed the experiments; K.J.S., A.J.P., S.P.Y. and A.S. designed the experiments; A.S. wrote the manuscript.

## Supplementary Material

Supplementary Information Supplementary information
