# Supplementary material for: A functional Kv1.2-hERG chimaeric channel expressed in Pichia pastoris
Source: Sci Rep. 2014 Feb 26;4:4201. doi: 10.1038/srep04201 (PMC3935203; doi:10.1038/srep04201)
Supplement: Supplementary Information [file srep04201-s1.pdf]

Supplementary Information

A functional Kv1.2-hERG chimaeric channel expressed in *Pichia pastoris*

Mandeep S Dhillon[1], Christopher J Cockcroft[1], Tim Munsey[1] Kathrine J Smith[3], Andrew J Powell[3], Paul Carter, David C Wrighton[1], Hong-lin Rong[1], Shahnaz P Yusaf[3] and Asipu Sivaprasadarao*[1,2]

School of Biomedical Sciences, Faculty of Biological Sciences, Multidisciplinary Cardiovascular Research Centre, University of Leeds, LS2 9JT, Leeds, U.K.

[3]GlaxoSmithKline, Gunnels Wood Road, Stevenage, Hertfordshire, SG1 2NY, UK

*To whom correspondence should be addressed: Asipu Sivaprasadarao, School of Biomedical Sciences, Faculty of Biological Sciences, University of Leeds, LS2 9JT, Leeds, U.K. Tel: +44-0113-3434326 Fax: +44-0-113-3434228; E-mail: a.sivaprasadarao@leeds.ac.uk

Methods associated with Supplementary Figure 2:

a) *Immunodetection of surface expressed S1-S6 chimaera in HEK cells***:** HEK-293 cells, grown on coverslips were transfected with S1-S6 chimaera in the pCDNA3 vector using Fugene® 6 (Roche) and stained as described previously [1]. Briefly, cells were fixed with 2% paraformaldehyde and stained using a rabbit antibody that recognises the surface exposed epitope of hERG (Sigma, antiKV11.1, K0640) and Cy3-conjugated secondary antibodies (Jackson ImmunoResearch). Images were acquired using Zeiss 510-META laser scanning microscope under an oil immersed 63x objective lens (NA = 1.4), using excitation and emission wavelengths of 550 nm and 570 nm.

b) *Western blotting***:** HEK293 cells were transfected with the S1-S6 chimaera in the pCDNA3 vector using Fugene® 6 (Roche). Three days later, membranes were prepared from transfected HEK293 cells and subjected to western blotting using anti-His-tag antibody (1:20000), goat anti-mouse-HRP conjugated IgG (1:40000), and SuperSignal West Femto Maximum Sensitivity ECL Substrate (Pierce).

Reference:

1 Taneja, T. K. *et al.* Sar1-GTPase-dependent ER exit of K_{ATP} channels revealed by a mutation causing congenital hyperinsulinism. *Human molecular genetics* **18**, 2400-2413 (2009).

Supplemental Figure 1

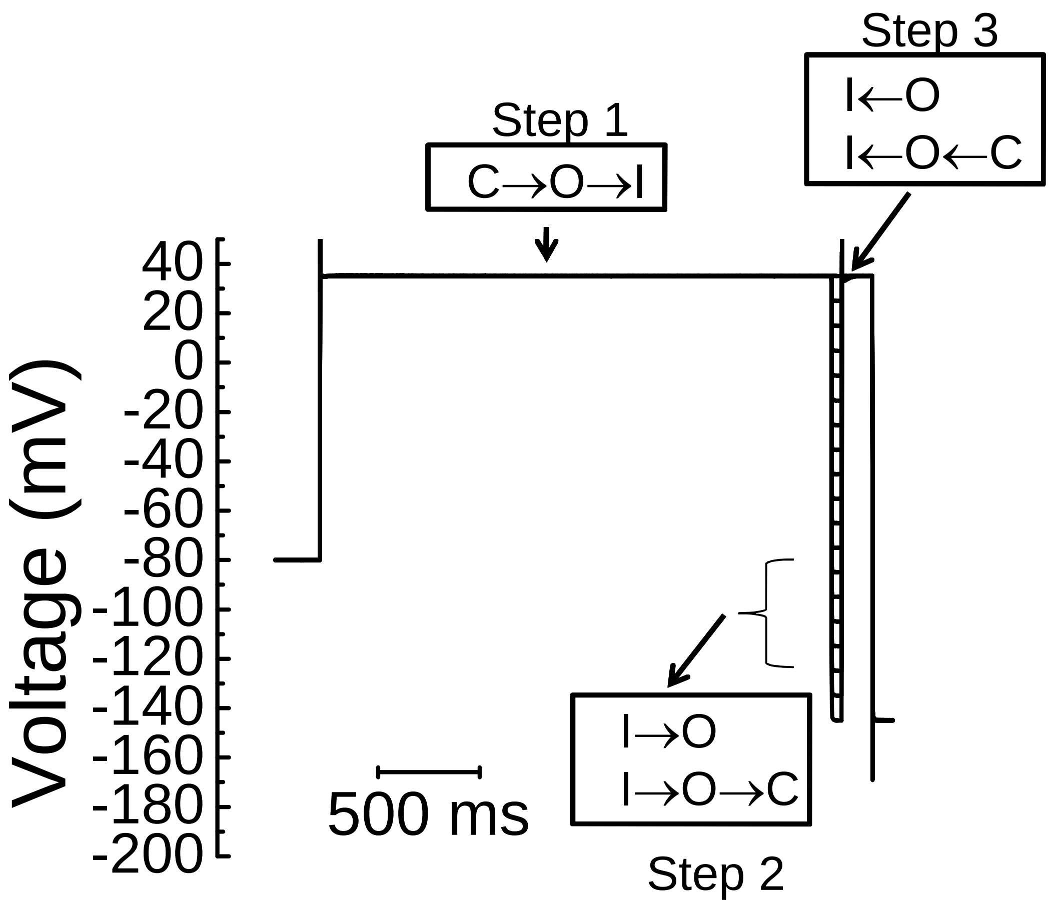

Supplementary Figure 1. Proposed gating events that lead to the Cole-Moore like shift. Proposed gating events that lead to the Cole-Moore like shift are depicted for different voltage steps during the three step protocol; C = closed; O = Open; I = inactivated. Unlike hERG, at hyperpolarising voltages in Step 2, some of the chimaeric channels enter the closed state due to relatively faster deactivation. The closed channels open slowly and undergo rapid inactivation leading to the Cole-Moore like behavior.

Supplemental Figure 2

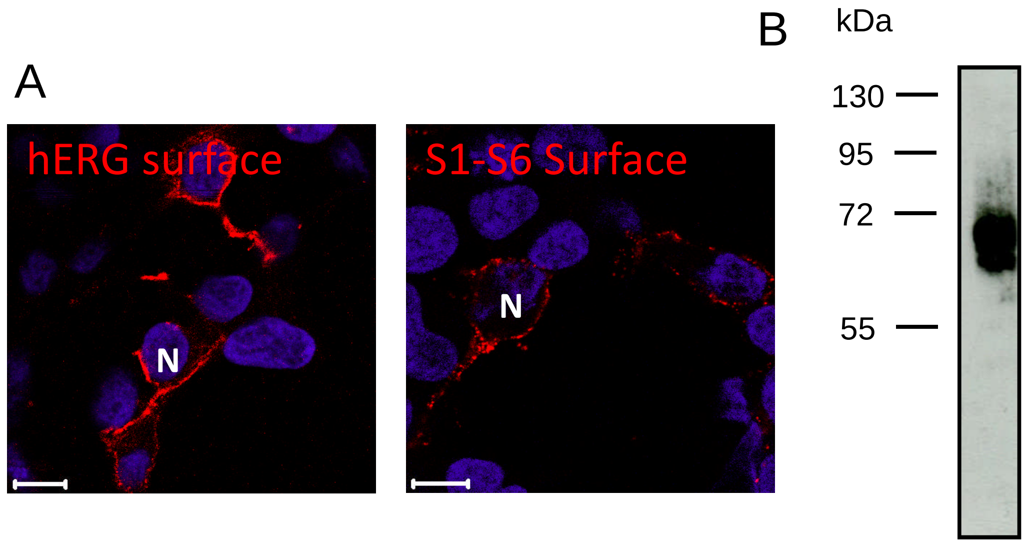

Supplementary Figure 2. Expression of the S1-S6 chimaera in HEK cells. (a) HEK-293 cells transfected with hERG or the S1-S6 chimaera in pCDNA3 were fixed and stained with anti-Kv11.1 antibodies that recognise the surface exposed epitope in the S1-S2 loop using a method described previously [1]. Surface staining indicates trafficking of the chimaera to the cell surface. Scale bars shown represent 10 µm; N represents the nucleus (blue, DAPI stained) of HEK 293 cells. (b) Western blot of crude membranes prepared from HEK293 cells transfected with pCDNA3-S1-S6; the two bands presumably correspond to core- and fully glycosylated S1-S6 chimaeric proteins.
